# The relationship between lateral cervical lymph node positivity rate and recurrence after comprehensive treatment in differentiated thyroid carcinoma: a single-center retrospective cohort study from China

**DOI:** 10.3389/fonc.2025.1484002

**Published:** 2025-02-13

**Authors:** Ting Ye, Shihan Shao, Shulin Yao, Ruimin Wang

**Affiliations:** ^1^ Department of Nuclear Medicine, The First Medical Center, Chinese People's Liberation Army (PLA) General Hospital, Beijing, China; ^2^ Department of Hepatobiliary Surgery, The Sixth Medical Center, Chinese PLA General Hospital, Beijing, China

**Keywords:** differentiated thyroid carcinoma, lateral cervical lymph node positivity rate, post-treatment recurrence, association, structural incomplete response, iodine-131 therapy

## Abstract

**Introduction:**

The lateral cervical lymph node positivity rate has been hypothesized to correlate with the recurrence risk in differentiated thyroid carcinoma (DTC) patients. However, the extent of this association within the Chinese population remains understudied. This study seeks to elucidate the potential causal link between the lymph node positivity rate and DTC recurrence.

**Methods:**

We conducted a retrospective cohort study, examining clinical records of 4,731 DTC patients who received surgical treatment at the First Medical Center of the General Hospital of the Chinese People’s Liberation Army from January 2015 to May 2020. The study variables encompassed demographic and clinical characteristics, including sex, age, tumor size, location, laterality, capsular invasion, lymph node metastasis counts, lymph node positivity rates, histological subtypes, Hashimoto’s thyroiditis co-occurrence, and the timing of iodine-131 therapy post-surgery. After applying strict inclusion criteria, 1,074 patients were selected for analysis. Recurrence was defined as structural incomplete response (SIR), confirmed by imaging or histological means. The lymph node positivity rate was calculated as the proportion of positive lymph nodes to the total lymph node count.

**Results:**

Multivariate analysis revealed a nonlinear association between the lateral cervical lymph node positivity rate and post-treatment recurrence, with a significant threshold at 0.5. The recurrence risk was substantially elevated with a positivity rate below this threshold (HR: 27.48, 95% CI: 7.21–104.70, P<0.0001), while no significant association was observed above it (HR: 0.17, 95% CI: 0.02–1.57, P=0.119). Subgroup analysis within the high-risk cohort did not yield a significant association between the positivity rate and recurrence risk (HR=0.43, 95% CI: 0.10–1.79, P=0.246).

**Discussion:**

In conclusion, this study identifies a nonlinear relationship between the lateral cervical lymph node positivity rate and the risk of DTC recurrence post-treatment. A positivity rate of less than 0.5 is positively associated with recurrence, while this association diminishes in significance among high-risk patients. This differs from the results previously reported. Further studies are needed to determine the potential mechanisms of the associations observed in observational studies.

## 1 Introduction

In recent years, the incidence of thyroid cancer has been increasing globally, ranking as the 9th most common cancer in 2018 (1). A similar trend is observed in China, where thyroid cancer now ranks 7th among all malignant tumors and 3rd among female malignancies, following breast cancer and lung cancer (2). Differentiated thyroid carcinoma (DTC) accounts for the majority of thyroid cancer cases. Although DTC is considered to have low aggressiveness, its 5-year mortality rate in China can exceed 10% (2). Patients experiencing structural recurrence after treatment face an even higher risk of mortality (3).

Lymph node metastasis is the most common and critical mode of spread in DTC, with a reported incidence of 20-50%, which is associated with postoperative recurrence (4–6). Notably, lateral cervical lymph node metastasis is linked to a heightened recurrence rate after treatment (7–9). Currently, the ATA risk stratification system is primarily used to predict the risk of recurrence post-treatment (10). Although this system includes factors such as histological subtype, extrathyroidal invasion, lymph node size, and number of positive lymph nodes, there remains considerable variability in treatment response among DTC patients within the same risk category. Recent studies have suggested that the positivity rate of lateral cervical lymph nodes may also correlate with the risk of recurrence following treatment in DTC patients (11–15).

To elucidate the relationship between the lateral cervical lymph node positivity rate and recurrence in differentiated thyroid carcinoma among the Chinese population, we conducted a retrospective cohort study aimed at clarifying this association.

## 2 Methods

### 2.1 Study design and study population

We conducted a retrospective analysis utilizing the electronic medical record system of the First Medical Center of the PLA General Hospital. Clinical data were collected from 4,731 consecutive patients diagnosed with differentiated thyroid carcinoma who underwent surgical intervention between January 2015 and May 2020. The data collected included demographic information such as sex and age, tumor characteristics like maximum diameter and location, the presence of single or multiple lesions, capsular invasion, the number of central neck lymph node metastases, the number of lateral neck lymph node metastases, positivity rates for central and lateral neck lymph nodes, pathological type, the presence of concomitant Hashimoto’s thyroiditis, and the interval between surgery and radioactive iodine (¹³¹I) treatment (hereafter referred to as the surgical-iodine interval). Outcomes were subsequently followed up for these patients after treatment. All patients provided informed consent prior to treatment, and this study was approved by the hospital’s ethics committee.

### 2.2 Inclusion criteria

All patients underwent total thyroidectomy with central and lateral neck lymph node dissection; Pathological diagnosis must be confirmed as differentiated thyroid carcinoma; Patients met the criteria for ¹³¹I treatment and subsequently received ¹³¹I therapy; Postoperatively, patients received TSH suppression therapy, maintaining treatment except during the peri-radioactive iodine period when therapy was temporarily halted; Patients demonstrated high compliance and were able to complete follow-up evaluations.

### 2.3 Exclusion criteria

Presence of other significant comorbidities that could adversely affect survival (e.g., other malignancies or severe trauma); Incomplete primary clinical data (e.g., unclear documentation of lymph node metastasis); Loss to follow-up during the study period.

**Figure 1:**
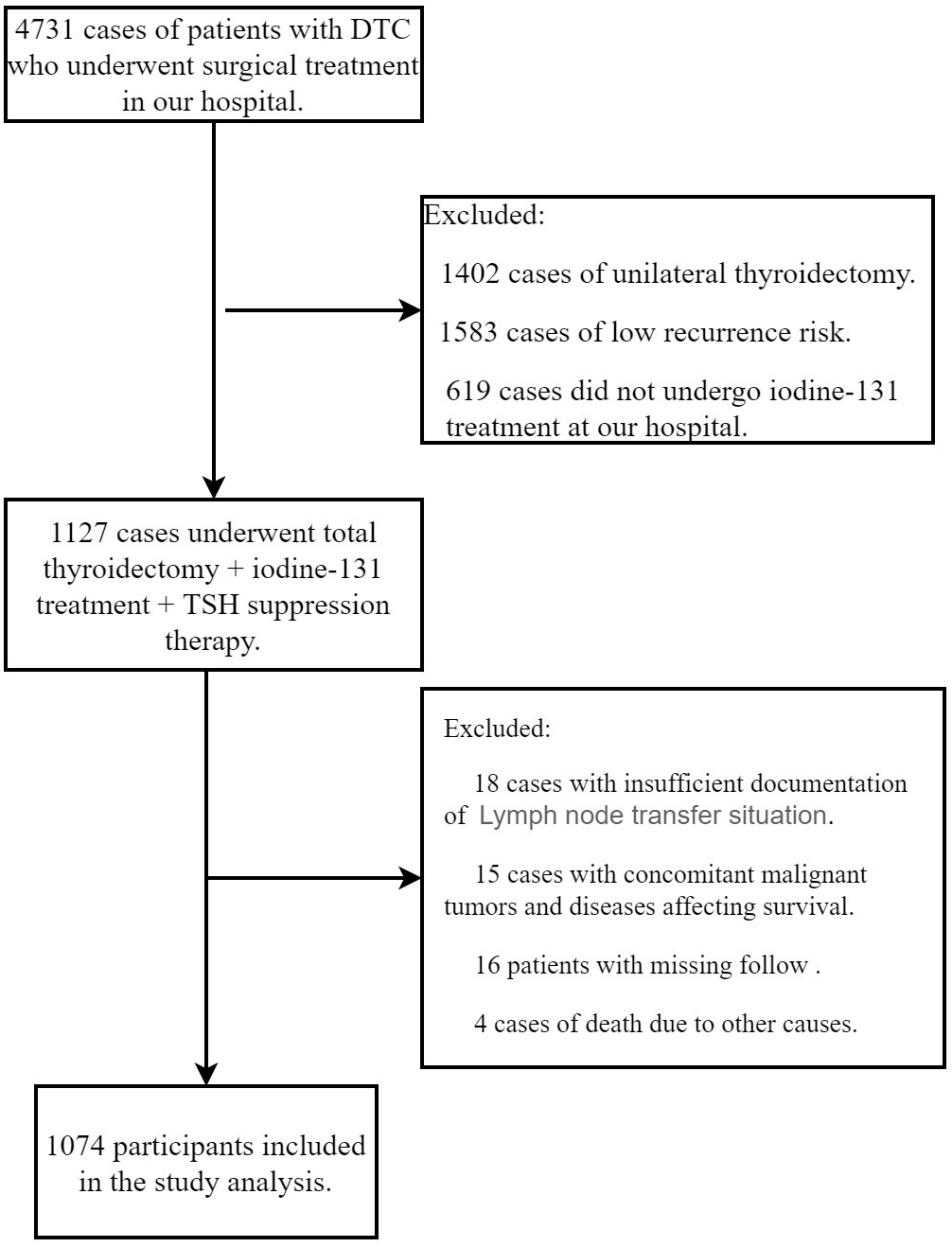
Flowchart of the study population.

This study received approval from the hospital’s ethics committee. After applying the inclusion and exclusion criteria, 1,074 subjects remained for data analysis from the original cohort of 4,731 participants (**Figure 1**).

All study patients underwent total thyroidectomy with central and lateral neck lymphadenectomy, followed by radioactive iodine (¹³¹I) ablation within 12 months post-operation. Prior to iodine treatment, patients adhered to a low-iodine diet (<50 µg/day) for 2 to 4 weeks, and any iodine-containing contrast agents or medications were avoided. L-T4 was discontinued for at least 2 to 4 weeks before iodine treatment to elevate serum TSH levels to >30 mU/L. A fixed dose of 150 mCi was administered for the single iodine treatment (10). Postoperatively, patients commenced TSH suppression therapy (excluding the 2 to 4 weeks prior to the initial iodine treatment), adhering to the suppression standards outlined in relevant guidelines (10).

The first follow-up evaluation was conducted 3 to 6 months after iodine treatment, with routine clinical assessments performed annually in the outpatient setting. These assessments included cervical ultrasound, chest CT scans, measurement of serum free thyroxine (fT4), thyrotropin (TSH), thyroglobulin (Tg), and anti-thyroglobulin (anti-Tg) antibody concentrations. Additionally, whole-body scans were performed 3 to 4 days post-iodine treatment to evaluate focal or diffuse uptake in the thyroid bed, cervical lymph nodes, and other sites. Any lesions suggestive of local recurrence were assessed via fine-needle aspiration cytology or imaging studies. Follow-up was conducted through telephone calls, text messages, and outpatient visits, starting from the date of iodine-131 treatment and concluding on December 31, 2021, resulting in follow-up durations of 18 to 81 months, with a median follow-up time of 43 months. Follow-up data were independently evaluated by two experienced attending physicians, and consensus was reached in case of any discrepancies.

Recurrence was defined as structural incomplete response (SIR), with the standard for determination based on imaging or histological findings, regardless of serum Tg levels (10, 16) The lateral lymph node positivity rate was calculated as the number of positive lymph nodes divided by the total number of harvested lymph nodes (12).

### 2.4 Statistical analysis

Continuous variables are expressed as means ± standard deviation (for normally distributed data) or medians (with minimum-maximum values) for skewed distributions. Categorical variables are presented as frequencies or percentages. To assess any statistical differences in means and proportions among groups, one-way ANOVA (for normally distributed data), Kruskal-Wallis H test (for skewed distributions), and Chi-square tests (for categorical variables) were employed. A univariate Cox proportional hazards regression model was utilized to evaluate the association between the rate of positive lateral lymph nodes and recurrence. Both unadjusted and multivariable-adjusted models are presented in this study. In accordance with STROBE guidelines, we simultaneously displayed the results of unadjusted, minimally adjusted, and fully adjusted analyses. Whether covariates were adjusted was determined by the principle that the odds ratio changed by at least 10% upon inclusion in the model (17). Additionally, we employed generalized additive models (GAM) to identify any nonlinear relationships. If a nonlinear association was observed, segmented linear regression models were conducted to calculate the threshold effect of the rate of positive lateral lymph nodes on recurrence based on the smoothed graphs. When the odds ratio between the rate of positive lateral lymph nodes and recurrence was significant on the smoothed curve, a recursive method automatically calculated the inflection point, and maximum likelihood estimation was used at the inflection point (18). Subgroup analyses were performed using stratified linear regression models. Likelihood ratio tests were utilized to evaluate modifications and interactions within subgroups. All statistical analyses were conducted using EasyFit statistical software (www.empowerstats.com) and R software. A p-value of less than 0.05 (two-tailed) was considered statistically significant.

## 3 Results

### 3.1 Participant selection

Out of 4,731 participants, 3,657 were excluded from this study. Among the 3,657 excluded subjects, 1,402 underwent unilateral thyroidectomy, 1,583 were classified as low recurrence risk, 619 did not receive ^131I treatment at our hospital, 18 had unclear records of lymph node metastasis, 15 had comorbid malignancies or diseases affecting survival, 16 were lost to follow-up, and 4 died due to other causes. Consequently, a total of 1,074 subjects were included for data analysis.

### 3.2 Baseline characteristics of patients

There were no statistically significant differences between patients with recurrence and those without recurrence regarding length of hospital stay, interval between surgery and ^131I treatment, sex, tumor location, capsular invasion, histological type, and comorbidities. However, statistical differences were observed in age, primary lesion size, central zone positive lymph node ratio, lateral zone positive lymph node ratio, whether the lesions were single or multiple, the number of central zone positive lymph nodes (≥5), the number of lateral zone positive lymph nodes (≥5), and recurrence risk stratification in **Table 1**.

**Table 1:** Characteristics of the study patients (n=1074).

Variable	Non-recurrent patients	Recurrent patients	P-value
N	975	99	
sex			0.272
Female	654 (67.08%)	61 (61.62%)	
Male	321 (32.92%)	38 (38.38%)	
Age (years)	43.40 (11.24)	40.65 (12.16)	0.021
Days in hospital (days)	8.44 (3.46)	8.95 (3.32)	0.159
titRAl (months)	3.51 (1.73)	3.35 (1.73)	0.378
Tumor size (cm)	1.10 (0.10-7.00)	1.50 (0.20-7.00)	<0.001
positive rate of central lymph nodes	0.33 (0.00-1.00)	0.75 (0.00-1.00)	<0.001
positive rate of lateral lymph nodes	0.00 (0.00-1.00)	0.25 (0.00-1.00)	<0.001
single/multiple lesions			0.021
single	433 (44.41%)	32 (32.32%)	
multiple	542 (55.59%)	67 (67.68%)	
tumor location			0.338
Isthmus	4 (0.41%)	0 (0.00%)	
single	548 (56.21%)	49 (49.50%)	
double	423 (43.39%)	50 (50.50%)	
presence or absence of capsular invasion			0.418
penetration	688 (70.56%)	66 (66.67%)	
presence	75 (7.69%)	6 (6.06%)	
absence	212 (21.74%)	27 (27.27%)	
number of positive central cervical lymph nodes			<0.001
≦5	878 (90.05%)	69 (69.70%)	
>5	97 (9.95%)	30 (30.30%)	
number of positive lateral cervical lymph nodes			<0.001
≦5	909 (93.23%)	74 (74.75%)	
>5	66 (6.77%)	25 (25.25%)	
pathological type			0.311
Papillary carcinoma	820 (84.10%)	86 (86.87%)	
Classic subtype	87 (8.92%)	10 (10.10%)	
others	68 (6.97%)	3 (3.03%)	
presence or absence of HT			0.334
no	746 (76.51%)	80 (80.81%)	
yes	229 (23.49%)	19 (19.19%)	
risk stratification			<0.001
Moderate risk	822 (84.31%)	59 (59.60%)	
High risk	153 (15.69%)	40 (40.40%)	

Results in the table: Mean(SD)/Median (Min-Max)/N(%).

P-value: For continuous variables, obtained using the Kruskal-Wallis rank-sum test; for count variables with theoretical counts <10, obtained using Fisher’s exact probability test.

the time interval from total thyroidectomy to RAI therapy (titRAI); Hashimoto’s thyroiditis (HT).

### 3.3 Univariate analysis

The results of the univariate analysis are presented in **Table 2**. The findings indicate that age, primary lesion size, central zone positive lymph node ratio, lateral zone positive lymph node ratio, presence of single/multiple lesions, the number of central zone positive lymph nodes (≥5), the number of lateral zone positive lymph nodes (≥5), and recurrence risk stratification were significantly associated with recurrence following comprehensive treatment. Conversely, variables such as sex, length of hospital stay, interval between surgery and ^131I treatment, tumor location, capsular invasion, histological subtype, and comorbidities were not associated with recurrence after comprehensive treatment.

**Table 2:** Univariate analysis.

Exposure	HR (95%CI)	P
sex		
Female	1.00	
Male	1.26 (0.84, 1.90)	0.257
Age (years)	0.98 (0.96, 0.99)	0.025
Days in hospital (days)	1.03 (0.98, 1.08)	0.287
titRAl (months)	0.95 (0.84, 1.07)	0.358
Tumor size (cm)	1.46 (1.26, 1.69)	<0.0001
single/multiple lesions		
single	1.00	
multiple	1.68 (1.10, 2.56)	0.016
tumor location		
Isthmus	1.00	
single	1074717.29 (0.00, Inf)	0.994
double	1410534.93 (0.00, Inf)	0.994
presence or absence of capsular invasion		
penetration	1.00	
presence	0.82 (0.36, 1.89)	0.644
absence	1.22 (0.78, 1.91)	0.387
number of positive central cervical lymph nodes		
≦5	1.00	
>5	3.50 (2.28, 5.38)	<0.0001
number of positive lateral cervical lymph nodes		
≦5	1.00	
>5	4.01 (2.55, 6.31)	<0.0001
positive rate of central lymph nodes	3.24 (1.95, 5.39)	<0.0001
positive rate of lateral lymph nodes	8.96 (5.00, 16.06)	<0.0001
pathological type		
Papillary carcinoma	1.00	
Classic subtype	1.19 (0.62, 2.30)	0.599
others	0.45(0.14, 1.41)	0.168
presence or absence of HT		
no	1.00	
yes	0.78 (0.47, 1.29)	0.331
risk stratification		
Moderate risk	1.00	
High risk	3.53 (2.36, 5.28)	<0.0001

Data in the table: HR (95% CI) P-value.

Outcome variable: SIR.

Exposure variables: sex; Age; Length of hospital stay; titRAl; Size of primary lesion; Single/multiple; Tumor location; Capsular invasion; Number of positive central lymph nodes; Number of positive lateral lymph nodes; Positive rate of central lymph nodes; Positive rate of lateral lymph nodes; Pathological type; Comorbidities; Risk stratification.

Adjusted variables: None.

Cox model time variable: Follow-up time.

### 3.4 Association between lateral zone positive lymph node ratio and recurrence

We utilized a univariate linear COX regression model to evaluate the relationship between the lateral zone positive lymph node ratio and recurrence. The findings are presented in **Table 3**, including both unadjusted and adjusted models. In the crude model, a positive association was observed between the lateral zone positive lymph node ratio and recurrence (HR=8.96, 95% confidence interval (CI): 5.00-16.06, P<0.001). In the minimally adjusted model (controlling for age and sex), the results remained largely unchanged (HR=8.25, 95% CI: 4.55-14.96, P<0.001). This association was still detected in the fully adjusted model (HR=4.02, 95% CI: 1.87-8.64, P=0.0004). For sensitivity analysis, we also treated the lateral zone positive lymph node ratio as a categorical variable (binary classification); however, no association was detected in the fully adjusted model when the ratio was >0.5 (HR=1.28, 95% CI: 0.72-2.28, P=0.405).

**Table 3:** Multiple regression equations.

Exposure	Non-adjusted	Adjust I	Adjust II
positive rate of lateral lymph nodes	8.96 (5.00, 16.06) <0.00001	8.25 (4.55, 14.96) <0.00001	4.02 (1.87, 8.64) 0.0004
≤0.5	1.0	1.0	1.0
>0.5	2.83 (1.70, 4.71) 0.00007	2.62 (1.56, 4.39) 0.0003	1.28 (0.72, 2.28) 0.405

Data in the table: HR (95% CI) P-value.

Outcome variable:SIR.

Exposure variable: Positive rate of lateral lymph nodes.

Non-adjusted model adjust for: None.

Adjust I model adjust for: sex; Age.

Adjust II model adjust for: sex; Age; Size of primary lesion; Single/multiple; Number of positive central lymph nodes; Number of positive lateral lymph nodes; Positive rate of central lymph nodes; Risk stratification; Length of hospital stay; titRAl; Tumor location; Capsular invasion; Pathological type; Comorbidities.

Cox model time variable: Follow-up time.

### 3.5 Analysis of nonlinear relationships

**Figure 2:**
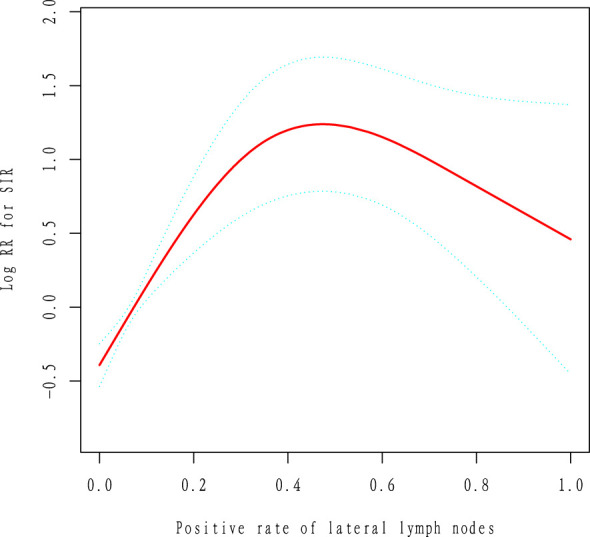
Smooth Curve Fitting SIR vs. Positive rate of lateral lymph nodes; Outcome: SIR; Exposure: Positive rate of lateral lymph nodes; Cox model time variable: Follow-up time.

Given that the lateral zone positive lymph node ratio is a continuous variable, it is essential to analyze potential nonlinear relationships. In this study (**Figure 2**), we identified a nonlinear relationship between the lateral zone positive lymph node ratio and recurrence following comprehensive treatment, after adjusting for sex, age, primary lesion size, single/multiple lesions, the number of central zone positive lymph nodes, the number of lateral zone positive lymph nodes, central zone positive lymph node ratio, length of hospital stay, interval between surgery and ^131I treatment, tumor location, capsular invasion, histological subtype, comorbidities, and risk stratification. Utilizing two piecewise linear regression models, we calculated the inflection point to be 0.5. To the left of this inflection point, we found the hazard ratio (HR), 95% confidence interval (CI), and P value to be as follows: HR=27.48, 95% CI: 7.21-104.70, P<0.0001. However, no relationship between the lateral zone positive lymph node ratio and recurrence was observed to the right of the inflection point (HR=0.17, 95% CI: 0.02-1.57, P=0.119) (**Table 4**).

**Table 4:** The results of two-piecewise linear regression model.

Inflection Point of Positive Rate of Lateral Lymph Nodes	HR	95%CI	P value
≤0.5	27.48	7.21 to104.70	<0.0001
>0.5	0.17	0.02 to 1.57	0.119

Data in the table: HR (95% CI) P-value.

Outcome variable: SIR.

Exposure variable: Positive rate of lateral lymph nodes.

Adjusted variables: sex; Age; Size of primary lesion; Single/multiple; Number of positive central lymph nodes; Number of positive lateral lymph nodes; Positive rate of central lymph nodes; Risk stratification; Length of hospital stay; titRAl; Tumor location; Capsular invasion; Pathological type; Comorbidities.

Cox model time variable: Follow-up time.

### 3.6 Subgroup analysis results

As shown in **Table 5**, the interaction test indicated a significant association with risk stratification (interaction P<0.0001). In contrast, the interaction tests for age, sex, primary lesion size, single/multiple lesions, capsular invasion, tumor location, and co-occurrence of Hashimoto’s disease were not statistically significant (interaction P values were 0.293, 0.438, 0.789, 0.544, 0.217, 0.322, and 0.746, respectively). We observed an interaction between the lateral zone positive lymph node ratio and risk stratification, revealing that the relationship between the lateral zone positive lymph node ratio and recurrence differed significantly across various risk strata. In patients with moderate recurrence risk, the lateral zone positive lymph node ratio was positively correlated with recurrence (HR=13.61, 95% CI: 5.68-32.57). However, in patients with high recurrence risk, there was no significant association between the lateral zone positive lymph node ratio and recurrence (HR=0.43, 95% CI: 0.10-1.79, P=0.246).

**Table 5:** Effect size of positive rate of lateral lymph nodes on recurrence in pre-specified and exploratory subgroups.

Variable	N	HR (95%CI) P	P for interaction
sex			0.293
Female	715	2.42 (0.80, 7.33) 0.118	
Male	359	5.48 (1.86, 16.20) 0.002	
Age(years)			0.438
<=55	931	3.77 (1.66, 8.56) 0.002	
>55	143	11.49 (0.80, 165.40) 0.073	
Tumor size group			0.789
Low	337	3.63 (0.25, 52.21) 0.343	
Middle	310	2.36 (0.49, 11.26) 0.282	
High	427	4.45 (1.69, 11.75) 0.003	
single/multiple lesions			0.544
single	465	2.80 (0.62, 12.59) 0.180	
multiple	609	4.76 (1.95, 11.65) 0.0006	
presence or absence of capsular invasion			0.217
penetration	754	3.00 (1.15, 7.81) 0.025	
presence	81	36.14 (2.25, 579.75) 0.011	
absence	239	5.47 (1.68, 17.84) 0.005	
tumor location			0.322
Isthmus	4		
single	597	3.60 (1.39, 9.34) 0.008	
double	473	7.19 (2.70, 19.11) <0.0001	
risk stratification			<0.0001
Moderate risk	881	13.61 (5.68, 32.57) <0.0001	
High risk	193	0.43 (0.10, 1.79) 0.246	
presence or absence of HT			0.746
no	826	4.62 (1.97, 10.85) 0.0004	
yes	248	3.35 (0.57, 19.55) 0.180	

Note 1: The above models adjusted for sex; age; size of primary lesion; single/multiple; number of positive central lymph nodes; number of positive lateral lymph nodes; positive rate of central lymph nodes; risk stratification; length of hospital stay; time interval between surgery and iodine-131 treatment; tumor location; capsular invasion; pathological type; comorbidities. Note 2: In each case, the model did not adjust for stratification variables.

## 4 Discussion

This study investigated the relationship between the positive lymph node ratio in the lateral neck region and recurrence after comprehensive treatment in a Chinese population with differentiated thyroid cancer (DTC). The fully adjusted model and sensitivity analysis showed that a lateral zone lymph node positive ratio greater than 0.5 was not associated with recurrence. However, a nonlinear relationship was identified, with the ratio positively correlating with recurrence on the left side of the inflection point (0.5) but not on the right. Notably, in patients with moderate recurrence risk, the lateral zone lymph node positive ratio was positively associated with recurrence, while this association was absent in those with high recurrence risk.

Our findings indicate that the positive lymph node ratio in the lateral neck region is associated with recurrence following comprehensive treatment in the Chinese population, which is partially consistent with previous studies but also shows some discrepancies (11–15). A comparative analysis reveals several differences between our study and prior research: first, the selection of study participants varied, as previous studies predominantly focused on patients with TNM staging, whereas our participants were those with moderate to high recurrence risk following total thyroidectomy. Furthermore, while earlier studies employed regression analyses to explore linear relationships, they did not examine potential nonlinear relationships. Lastly, subgroup analyses are crucial in scientific research (19). Unfortunately, the aforementioned studies used only sex and age as stratification factors for subgroup analyses and did not perform interaction tests, impeding a comprehensive exploration of the true relationship between the lateral zone lymph node positive ratio and recurrence.

In our study, we utilized various stratification variables including age, sex, tumor size, single/multiple lesions, tumor location, capsular invasion, risk stratification, and the presence of concomitant Hashimoto’s disease. We observed differing relationships between the lateral zone lymph node positive ratio and recurrence based on risk stratification. Compared to the high-risk group, the positive association between the lateral zone lymph node positive ratio and recurrence was more pronounced in the moderate risk group. This finding has not been previously reported. Analyzing the potential reasons, we believe that the positive lymph node ratio is closely related to the number of positive lymph nodes, with more than five positive nodes being an important criterion for inclusion in the moderate recurrence risk group. In contrast, the high recurrence risk group was categorized primarily based on the size of metastatic lymph nodes and extrathyroidal invasion, resulting in the absence of a association between the lateral zone lymph node positive ratio and recurrence.

Previous studies have indicated that chronic autoimmune thyroiditis (Hashimoto’s thyroiditis, HT), a prevalent thyroid disorder, is often associated with alterations in thyroid function and an increased risk of thyroid cancer (20, 21). HT may influence the development of papillary thyroid carcinoma by modifying the tumor microenvironment. Chronic inflammation is recognized as a significant risk factor for tumorigenesis, and the inflammatory response associated with HT may contribute to genetic mutations and the malignant transformation of thyroid cells (22). Furthermore, the potential infiltration of lymphocytes in the thyroid tissue of HT patients may create a supportive microenvironment for tumor cells, thereby facilitating tumor growth and metastasis (23). However, this phenomenon was not observed in our study.

Our study has several strengths. Firstly, we employed not only generalized linear models to evaluate the linear relationship between the lateral zone lymph node positive ratio and recurrence but also generalized additive models (GAM) to elucidate nonlinear relationships. GAM has distinct advantages in addressing nonlinear relationships, allowing for nonparametric smoothing and fitting regression splines to the data. The use of GAM will aid us in better elucidating the true relationship between exposure and outcomes. Secondly, this retrospective study incorporated unavoidable potential confounding factors; therefore, we applied rigorous statistical adjustments to minimize residual confounding. Despite previous research reporting a linear relationship between the lateral zone lymph node positive ratio and recurrence, we did not observe such a relationship in populations where the lateral zone lymph node positive ratio was greater than 0.5. Thirdly, the effect modification analysis enhanced the utility of the data, revealing that no such relationship was found between the lateral zone lymph node positive ratio and recurrence in the high-risk subgroup.

Although the current American Thyroid Association (ATA) guidelines do not yet incorporate the positive rate of cervical lymph nodes in differentiated thyroid cancer into recurrence risk stratification, our study further elucidates the relationship between the positive rate of lateral cervical lymph nodes in papillary thyroid carcinoma and recurrence following comprehensive treatment. As additional evidence-based medical data accumulates and the biological mechanisms linking lymph node positivity to recurrence risk are explored, more optimized recurrence risk assessment models are expected to be integrated into clinical practice. This will provide a solid foundation for postoperative monitoring and treatment strategies for patients.

However, our study has certain limitations. First, being an analytical retrospective study, it provides only weak evidence regarding the exposure-outcome relationship, making it challenging to distinguish causal relationships. Secondly, the study population comprised only Chinese individuals, which may limit the generalizability of the findings to other populations. Thirdly, data on genetic testing, particularly the status of BRAF mutations (24, 25), were not included in the study. Moreover, due to sample size limitations, certain pathology subtypes associated with a high risk of recurrence, such as high cellular variant, columnar cell variant, and tall-cell variant of papillary thyroid carcinoma (PTC), were not included in this study (26). Therefore, the conclusions of our study warrant further validation through clinical research with larger sample sizes and diverse populations.

## 5 Conclusion

There exists a nonlinear relationship between the lateral zone lymph node positive ratio and recurrence following comprehensive treatment. When the lateral zone lymph node positive ratio is less than 0.5, it is positively correlated with recurrence. Additionally, no such association was observed among subjects in the high recurrence risk group.

## Data Availability

The raw data supporting the conclusions of this article will be made available by the authors, without undue reservation.
